# Supplementary figures and images for: A specific EMC subunit supports Dengue virus infection by promoting virus membrane fusion essential for cytosolic genome delivery
Source: PLoS Pathog. 2022 Jul 14;18(7):e1010717. doi: 10.1371/journal.ppat.1010717 (PMC9321775; doi:10.1371/journal.ppat.1010717)

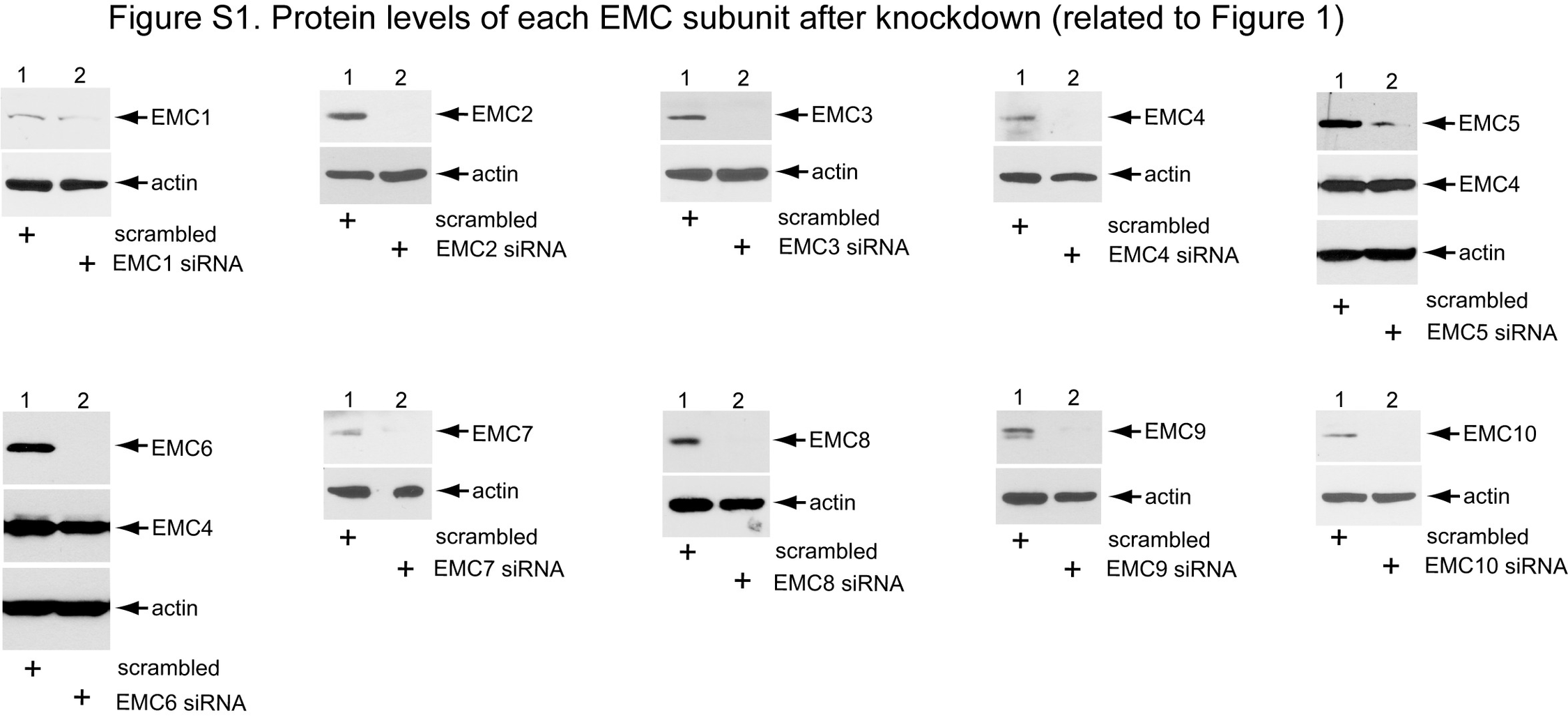

Supplement: S1 Fig — Extracts derived from HEK 293T cells transfected with the indicated siRNA were subjected to SDS-PAGE followed by immunoblotting using the indicated antibodies. (TIF) [file ppat.1010717.s001.tif]

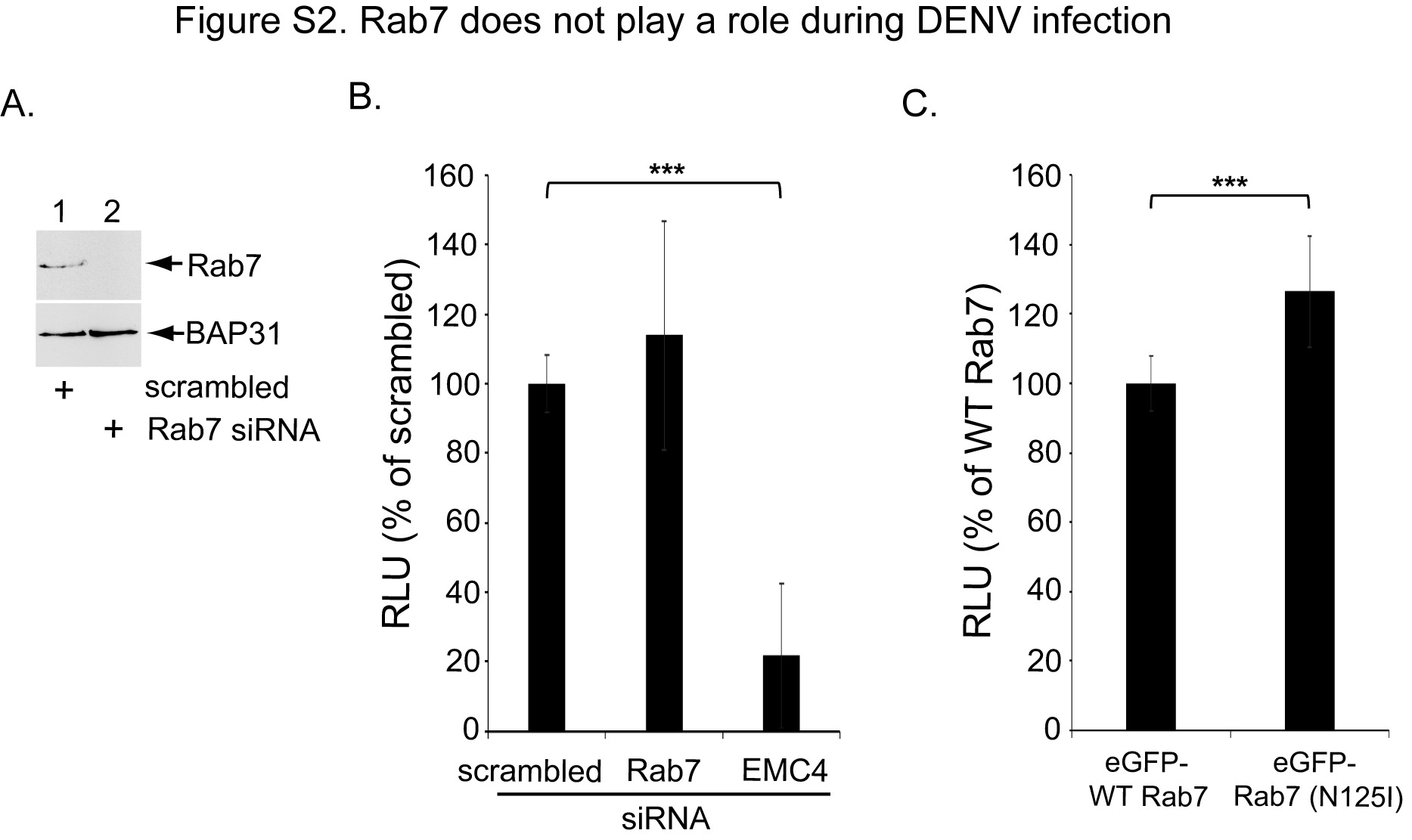

Supplement: S2 Fig — A. Huh 7.5.1 cells were transfected with the indicated siRNAs and lysed with 1% Triton X-100, and the resulting extract was subjected to SDS-PAGE and immunoblotted with the indicated antibodies. B. Huh 7.5.1 cells were transfected with the indicated siRNAs followed by 48 h infection with luc-DENV2 (MOI 0.05). The data show the relative luciferase unit normalized to the scrambled siRNA, and represent means and standard deviations (SD) (n ≥ 3). C. Huh 7.5.1 cells were transfected with the indicated plasmid followed by 48 h infection with luc-DENV2 (MOI 0.05). The data show the relative luciferase unit normalized to the WT Rab7, and represent means and standard deviations (SD) (n ≥ 3). ***P ≤ 0.001. (TIF) [file ppat.1010717.s002.tif]
