# Supplementary material for: A specific EMC subunit supports Dengue virus infection by promoting virus membrane fusion essential for cytosolic genome delivery
Source: PLoS Pathog. 2022 Jul 14;18(7):e1010717. doi: 10.1371/journal.ppat.1010717 (PMC9321775; doi:10.1371/journal.ppat.1010717)
Supplement: Attachment — Submitted filename: Bagchi and Speckhart et al. (Point-by-point Response).docx [file ppat.1010717.s003.docx]

**Major Issues: Key Experiments Required for Acceptance**

**Reviewer #1:**

Major points:
1. The reversal of the inhibition by EMC4 depletion using PS is a critical experiment. However, it is odd that this is the lipid that is used—the original ref 8 report proposed the LE lipid BMP as the key lipid, although both PS and PG could enhance DENV fusion with liposomes or with the plasma membrane. What is known about the lipid specificity that is needed for this effect in their system? Can BMP or PG substitute? How specific is the detection with lact-C2-GFP for PS vs. other acidic lipids? Given that this fluorescent probe shows considerable staining of the LE in the EMC4 depleted cells (Fig. 5A), it is important to address whether the depletion of EMC4 is actually acting via another acidic lipid. While the inhibition of PS-synthesis enzymes is helpful, further experiments that address the lipid specificity are important as well.

Response: We agree that testing the lipid specificity is important, and assessed if BMP participates in the EMC4-dependent DENV entry step. Specifically, we performed the EMC4 knockdown (KD)-rescue infection experiment with addition of exogenous BMP. Under the same conditions used in Fig. 5C, we found the block in DENV infection under EMC4 KD was not restored by adding BMP. However, we hesitate to draw any firm conclusion from these findings because it is possible that we have not found the optimal condition for the BMP-dependent rescue study. As such, we have toned down our conclusion in the revised manuscript (both in the discussion section and in the model) to indicate that EMC4 may assist the ER-to-LE transfer of other lipids during EMC4-dependent DENV entry.

Regarding the specificity of LacC2-GFP for PS vs other acidic lipids, the original paper describing this fusion protein (PMID: 18187657) extensively addressed this question and found that it specifically interacted with PS without binding to PC, PE, PI, PA, or PIP2.

2. It is crucial to prove that EMC4 depletion does not inhibit DENV binding or endocytic uptake but rather DENV fusion and RNA release. The evidence for the lack of effect on binding/ endocytosis is Fig. 3F. In order to be convincing, the linearity of this RT-qPCR assay vs virus amount bound and/or endocytosed should be included. This would also strengthen the assays in Fig. 3C and 3E. More experimental details should be included here (see point 4 below).

DENV2 RNA

| Log10 copy number | Ct value |
| --- | --- |
| 2 | 38.38 |
| 3 | 34.85 |
| 4 | 32.17 |
| 5 | 28.59 |
| 6 | 25.3 |
| 7 | 22.16 |
| 8 | 19.98 |
| 9 | 17.29 |

Response: We now synthesized *in vitro* transcribed DENV viral genome and used it to generate a standard curve (shown on the right) which revealed that the genome level used in our analysis of viral entry was within the linear range of this curve.

3. The authors find that siRNA depletion of rab7 does not reduce DENV infection (Fig. S2). This is taken as evidence that EMC4 does not require the previously observed binding to rab7 for its action in bridging LE-ER and transferring PS. The lack of effect on infection is puzzling since numerous studies have shown that DENV fuses in the LE, and delivery to this compartment requires rab7. The authors cite one paper in which rab7 depletion by siRNA inhibits DENV infection and comment that they don’t know the reason for the discrepancy. They do not mention the many other studies that found inhibition of DENV infection by dominant-negative rab7. The siRNA depletion looks convincing, but the result here would be strengthened by demonstrating that the depletion is sufficient to block the EMC4-rab7 interaction or downstream effects of that. What would happen with a DN-rab7? As it stands now, this result is not in sufficient depth to be convincing

Response: As requested, we now show that DENV infection is unaffected by expression of the N125I Rab7 dominant negative construct (Fig S2C).

4. Overall the paper is quite light on experimental details, which should be added to the legends and/or methods. For example, please indicate the times of siRNA transfection, times of infection, etc. In Fig 3D, please indicate what proportion of the total was loaded vs. the cytosol and endosome fractions. The use of rab7 as a LE marker is odd, since it cycles on and off the membrane/cytoplasm, and a LE protein like CD63 or LAMP1/2 would be a better choice.

Response: As requested, more experimental details are now included in the figure legends and the methods section. Additionally, we now generated new data using LAMP1 as a marker for the endosome fraction (Fig 3D), as suggested.

5. The end of the discussion is overstated. There is no evidence that the release of the vRNA from the LE at a site close to the ER would promote infection, although it could do so. Conversely though, fusion of the virus with the plasma membrane or transfection of cells with the vRNA is known to efficiently cause infection. The authors should tone down this argument.

Response: Thank you, we have changed the wording in the discussion.

**Reviewer #2:**

Major point:

One experiment is required to bring this study full circle. In ref. 8 in your manuscript, the authors could enhance fusion with exogenous PS and overcome the fusion defect in the absence of PS with chlorpromazine (CPZ), which resolves hemifusion intermediates. This should be tested in the context of EMC4 depletion. The PS addition would test whether EMC4 is required for LE PS, while complementation of DENV fusion/entry/replication by CPZ would link the two phenotypes. Some variation on this type of complementation experiment would greatly improve the manuscript.

Response: This is a good suggestion. We performed this experiment and found that under EMC4 KD, addition of CPZ at the reported effective concentration (> 1 ug/mL) (PMID: 20949067, PMID: 20122152) compromised cellular integrity. Addition at lower CPZ concentration (125 or 500 ng/mL) under EMC4 KD did not compromise cellular integrity – under these conditions, we found that the block in DENV infection due to EMC4 KD was not restored (see figure to the right). However, we are careful to draw any firm conclusion from these findings because these lower CPZ concentrations may not be effective in resolving the hemi-fusion intermediate.

**Reviewer #3:**

Major points:

1) Fig. 1A/S1: The authors chose to use siRNA KD of EMC subunits, which is hampered by variability of KD efficiency. Based on this experimental setup they conclude that EMC4 but not other subunits promotes dengue infection as measured using a Luciferase virus at 48h. This is contradictory to numerous other studies (e.g. Savidis et al. ( PMID: 27342126), Ngo et al. (PMID: 31516121)), which showed that all (tested) EMC subunits are critical for flavivirus infection. While the authors show that EMC4 siRNA treatment efficiently reduced EMC4 mRNA levels (~>95% reduction) as well as led to depletion of EMC4 protein, they did not show that other EMC subunits were sufficiently depleted in their experiment. In fact, mRNA levels were only reduced by 70-90%, which may not be sufficient to disrupt EMC function and could explain the lack of phenotype. Savidis et al showed a reduction of DENV replication for siRNAs against multiple EMC subunits, further supporting that the observation here is due to technical reasons. The authors should address this by either using more efficient siRNAs against other subunits and validation of protein depletion by Western Blot, or CRISPR KO/I methods.

Response: As requested, we now show the protein level of each EMC subunit after siRNA-mediated KD by Western blotting (Fig. S1). Our results demonstrate that the protein levels of each EMC subunit were efficiently depleted under the KD condition. It is important to note that a previous study found that KD of EMC4 did not affect the level of the other EMC subunits (PMID 30578317), supporting our central idea that EMC4 is specifically required during DENV-endosome membrane fusion essential for virus infection. We have now more carefully indicated this in the revision.

We do not believe our findings contradict the findings of Savidis et al. or Ngo et al. These studies found that chronic EMC depletion (72 hr siRNA treatment using a siRNA targeting a specific EMC subunit) led to a strong block in DENV infection. Because other EMC subunits are likely also depleted under this chronic knockdown condition, it is difficult to determine which specific EMC subunit is responsible for the phenotype. Instead, in our study, we performed acute EMC depletion (48 hr siRNA treatment using a siRNA targeting a specific EMC subunit) and surprisingly found a robust block in DENV infection only under EMC4 knockdown (Fig. 1A). Importantly, we demonstrate that acute EMC4 knockdown did not affect the level of other EMC subunits (Fig. 1D). Additionally, we found that acute depletion of the core EMC subunits EMC5 or EMC6 did not reduce the level of EMC4 (Fig. S1), consistent with the observation that EMC5 or EMC6 knockdown did not significantly block DENV infection.

2) The authors show that EMC4 siRNA selectively depletes EMC4 protein levels without affecting the expression of other subunits (Fig 1D). This is consistent with a previous report (PMID: 29809151) that showed that EMC4 CRISPRi did not reduce expression levels of other subunits, while EMC2 CRISPRi did. EMC4 may thus not be important for the overall structural integrity of the complex but no conclusion about the functionality of the complex can be drawn. Indeed, genetic interaction mapping and whole-proteome analysis of EMC2 and EMC4 CRISPRi KD experiments suggests that their depletion has similar molecular consequences.

Furthermore, the conclusion that EMC4 does not promote biogenesis of flaviviral NS proteins is not well supported. In Fig 2A the authors used expression of NS4B as a readout whether EMC4 is required for NS4B biogenesis. However, the Ngo et al study (PMID: 31516121) demonstrated that NS4B expression is impaired only in the context of the NS4A-NS4B polyprotein sequence (containing the ER-targeting 2K signal peptide). Additionally, that study used EMC4 KO cells and demonstrated a defect in viral protein biogenesis. The authors should thus repeat the experiment with an appropriate polyprotein construct. Additionally, to make EMC4-specific claims about a lack of effect on polyprotein biogenesis, the authors should also include validated KD of other EMC subunits for comparison.

Response: We apologize for the confusion. In addition to showing that acute EMC4 KD did not affect the expression of NS4B using a transfected plasmid encoding NS4B (Fig. 2A), we further showed (in Fig. 2B) using a DENV replicon system that acute depletion of EMC4 did not block NS4B expression. Additionally, using this replicon system, we demonstrate that EMC4 KD did not impair formation of the dsRNA intermediate (Fig. 2C/D) or viral genome replication (Fig. 2F).

3) Fig 2C-E: The authors should provide experimental details (timepoint of harvest after siRNA transfection) and control data to ensure that EMC4 protein levels were fully depleted. As the experimental system is a stable replicon cell line, additional controls (e.g. a replication inhibitor or an siRNA targeting a host factor known to affect viral genome replication (e.g. oligosaccharyl transferase) need to be included. Otherwise, it is possible that the observed lack of decrease of DENV RNA levels upon EMC4 treatment is due to the experimental setup, such as timepoint and readout (remaining EMC4 protein needs to be depleted, then viral RNA needs to be degraded. Even then qPCR is generally based on the amplification of short RNA fragments and may obscure an effect on the already present viral RNA). An additional readout on viral protein level should be performed.

Response: We agree that the use of an established DENV replication inhibitor in our replicon system is a good control. As requested, we now used the established DENV replication inhibitors (NITD008, an RNA polymerase inhibitor) in the replicon system and found that these inhibitors indeed potently blocked the dsRNA signal (Fig. 2E), in contrast to depletion of EMC4 (Fig. 2D). These control results support the validity of the DENV replicon system.

4) Fig 3: The experimental setup is not suitable to draw conclusions about early steps in dengue infection. Why do the authors think that differences in viral RNA measured at 48hpi are due to an entry defect? At this timepoint the virus has undergone multiple rounds of entry, translation, genome replication, assembly and egress. The conclusion seems to be based on comparison with the “negative” result from Fig 2E. To measure effects specifically on entry, the authors should collect cells within ~12 hpi, where no/little replication has occurred (ideally in presence of a polymerase inhibitor).

Response: We apologize for this confusion. Figs. 3A,B,C were not meant to show an EMC4-dependent effect at the early time point (i.e. entry defect) – these figures only demonstrate that EMC4 plays a role in DENV dsRNA formation (Fig. 3A/B) and genome replication (Fig. 3C). For these studies, we used 48 hpi to ensure that DENV replication has occurred. However, to pinpoint which specific step UPSTREAM of DENV replication EMC4 exerts a role, we instead used the 1 hpi time point to capture the early events (i.e. viral entry) (Figs. 3D, E, and F). At 1 hpi, we found that depletion of EMC4 blocked arrival of the DENV genome to the cytosol (Figs. 3D/3E) without impairing initial virus entry/endocytosis (Fig. 3F) – these results thus suggest that EMC4 participates in a step that delivers the viral genome into the host cytosol after initial endocytosis. I hope this clarifies the confusion.

5) As the authors make the claim that EMC4 supports DENV genome release into the cytosol, they should include KD/KO of another EMC subunit for comparison in the cellular fractionation and DiD labelling experiments. This would probe whether indeed EMC4 specifically or rather the entire complex is required for viral entry.

Response: As requested, we now show that KD of EMC3 in Huh7.5.1 cells (Fig. 4C) did not affect membrane fusion between DENV and the endosome membrane, as shown by the DiD labelling experiment (Fig. 4D and 4E).

6) Fig 5: The authors use EMC3 KD as a negative control based on the observation in Fig 1 that EMC3 is not important for dengue infection. However, this is contradictory to numerous other studies and may be due to insufficient KD. As mentioned above, KD should be validated on protein level or complete KO cells should be used.
To further support the role of PS in viral entry, the authors could utilize the fractionation assay and DiD assay.

Response: As requested, we now show by Western blotting that EMC3 is efficiently KD in the Huh7.5.1 cells (Fig. 4C). We now further show that EMC3 KD did not affect DENV-endosome membrane fusion (Fig. 4D and 4E). These findings support the idea that EMC4 plays a specific role in DENV-endosome membrane fusion essential for virus infection.

7) The authors should confirm EMC4 and PS dependency for viral entry using another flavivirus (e.g. Zika), for which EMC subunits were also identified as critical host factors. Additionally, the authors should include a negative control virus, which does not utilize late endosomes/EMC

Response: We in fact reported that Hepatitis C virus infection was not blocked when the entire EMC complex is depleted using EMC KO cells (PMID: 31067454), consistent with other reports (PMID: 27383987; PMID: 31516121). Additionally, as requested, we now show in Fig. 1E that EMC4 KD decreased Zika virus (ZIKV) infection.

**Minor Issues: Editorial and Data Presentation Modifications**

**Reviewer #1:**

Minor points:

1. On P17 of discussion and again at end of discussion please reword. The vRNA is not co-translationally translocated, the nascent polyprotein is.

Response: Thank you - we have changed the wording.

2. The model in Fig. 5F needs work. It should clearly show that the virus NC assembles on the cytoplasmic face of the ER and buds into the ER lumen, followed by transport of the virus particle in secretory vesicles.

Response: This model has been modified, as suggested.

**Reviewer #2:**

Minor points:

1. The authors normalize their control in all experiments to 100 without any error. This is problematic because it skews p values to be more significant than they are. The error in control samples should be maintained (include error bars) and p-values recalculated. P-values should then be included in figures.

Response: As requested, we have now included error bars in the controls and the p-values are added in the figures throughout.

2. In Fig. 2C & 3A, the dsRNA IF is difficult to appreciate. Could you use a higher magnification (5-10 cells visible)?

Response: We have now included a zoomed-in image, as requested.

**Reviewer #3:**

Minor points:

1) The authors wrote in the introduction: “To identify host factors that facilitate DENV infection, several groups used the genetic CRISPR/Cas9 loss-of-function approach….”
Please provide references to these studies (Zhang et al; Marceau et al; Ma et al; Savidis et al; Hoffmann et al). The statement may also be altered to include other flaviviruses that – like dengue – depend on EMC.

Response: We have included these references and changed the statement to “To identify host factors that facilitate flavivirus (including DENV) infection….), as suggested.

2) Can the authors provide more information on the quantification of the SIM experiment (how many FOVs/cells were quantified?)

Response: We have now provided this information in the Methods section.

3) As PTDSS1/2 are potential flavivirus host factors working in concert with EMC4, can the authors provide information how these genes scored in the CRISPR screens relative to EMC4? Is there activity (largely) redundant, which may preclude enrichment in a single gene KO CRISPR screen

Response: The Savidis et al study (PMID 27342126) did not find PTDSS1 or PTDSS2 in their flavivirus CRISPR study. It is possible that there is redundancy in their activity.
